# Supplementary material for: Factors influencing farming households’ climate change adaptation strategies in Central Vietnam
Source: PLoS One. 2025 Jul 10;20(7):e0328058. doi: 10.1371/journal.pone.0328058 (PMC12244606; doi:10.1371/journal.pone.0328058)
Supplement: Attachment — Submitted filename: Report to reviewers.docx [file pone.0328058.s003.docx]

**RESPONSE REPORT TO REVIEWERS**

The authors would like to thank the editor and reviewers so much for detailed comments and suggestions. These are very valuable comments that help the authors improve the article. The authors have fully received and revised the entire article according to the comments of the reviewers. Below is the author's detailed explanation and modification (in red text) according to each comment point of the editor and reviewers. We have also reviewed and improved the entire article in terms of rationale, content, model, academic references, language and format (Please see the revised draft for more detail). The authors hope to receive considerations by the editor and reviewers.

**Reviewer #1:**

With pleasure, I reviewed the article titled: "Factors Influencing Farming Households’ Climate Change Adaptation Strategies in Central Vietnam, ref: PONE-D-24-25204, which was submitted for publication in PLOS ONE Journal, it is both relevant and engaging. The paper was also pleasant to read.

While the paper is promising, there are some minor suggestions for improvement. If you don’t mind, I have included some comments below that I hope, will help enhance the overall quality of the manuscript. Please find them below:

*Please add a short title.

Thanks much for the suggestion! We want this to be a case study in Vietnam and the implications of the study can be referenced for other coastal areas in Vietnam that are also affected by natural disasters in the context of climate change. Therefore, we have chosen a broader title.

*Abstract
1. I suggest to write “most vulnerable to the impacts of climate change”.

We have corrected as reviewer comment!
2. I think you have to start your abstract by a more general background instead of writing directly about Vietnam.

We have added the general background as suggested. ‘Climate change is a huge challenge to human development in the 21st century’.

3. Please consider removing the part when you specify the software you used for your statistical analyses “Data were analyzed using SPSS 23.0 software”. However, you still have to indicate the method you followed and the analyses you have done.
We have deleted ‘using SPSS 23.0 software’ and only indicate the method for data analysis.
*Introduction
1. I really appreciated the way you started your introduction, well done. Please consider a general approach like this in your abstract.

Thanks so much! We have done as you suggestion in the Abstract (please see the revised paper).
2. Honestly, I am not really convinced by the end of your introduction, you explained the structure of the article; I don’t think it is necessary. Additionally, I think the main objective may be unclear for the readers. You explained the gap in current literature and the importance of your study. Nevertheless, it can be interesting to be clearer (eg. This study aimed at….the aim of the study was….etc.).

We deleted the final paragraph about structure of the paper as comment. We also have put a sentence to clarify the objective of the study as suggestion.
‘…This study aim at analysinig the factors that influence the choice of adaptive livelihood strategies of farming households in Vietnam..’

*Methods
1. Please add the GPS coordinates for the study area.

We have added GPS coordinates for the study area Tuyen Hoa district (17.7882° N, 106.2051° E).
2. The quality of Figure 1 looks a little poor. Please consider improving it. In addition, I saw source: (2022), did the authors obtain permission from Truong for the reuse of this material? Why not create your own map in that case? It would significantly enhance the quality of this manuscript.

We have replaced the Figure with a new one with higher resolution according to your suggestion.

3. The formula of your sample size should also be numbered.

We have numbered the formula as suggested.

4. I think you have to separate “data collection” from “statistical analysis”

We include the data collection and analysis in section 2.3 (Data collection and analysis).

5. Please add the full name of the software you used Statistical Package for the Social Sciences (SPSS) instead of the acronym, as it was the first time it appears in the text.

We have added and corrected as your suggestion
*Results and discussions
1. The quality of Figure 3 is also a little poor, please consider increasing its resolution

We have replaced Figure 3 with higher resolution
2. Please consider adding some references from 2023 and 2024, you don’t have to limit yourself to papers related to your geographical area, you can consider adding other works to support some statements.

We have added some references from 2023-2024 (in the text and the lists) to clarify arguments as comment.

3. Apart from that, the discussion section was well conducted, I congratulate the authors.
Thanks so much!

*Conclusion & Implication
1. I think this part is too long, please consider shortening it. The part related to limitations must be put in the end of the discussion section, not in the conclusion.

Thank you for your comment. We have shortened the Conclusion & Implication. We have also added limitations to the discussion section as suggested.
Finally, I would like to thank the authors for this interesting research; I wish them good luck.

Once again, the authors would like to thank the reviewer so much and hope to receive the consideration from the reviewer.

**Reviewer #2:**

To the Authors
I founded many written mistakes along the paper. I will mention some examples:
a. Competing interests: a T is missing when your response starts.

We have checked and corrected it as suggested.
b. '.... might reduce farming vulnerability and rikds to changes in the climate system'. Reduce (double space farming) and 'RIKDS' change to risks.

We have checked and corrected it as suggested.
c. Better sentence SYNTAX. From 'Especially, climate change is causing severe impacts on food production and economic development in the agricultural sector' TO 'Especially the food production and economic development in the agricultural sector is being impacted by Climate Change'.

We have checked and corrected it as suggested.
d. 'chains (Lien et al., 2018, Le and Nguyen, 2029).. However,'. Double full period. We have checked and corrected it as suggested.
e. SugGestion: replace 'quite a few' with 'several' for a formal writing.

We have checked and corrected it as suggested.
f. 'Identified factor groups included behavioral, psychological, or socio-economic factors of the farmers.' Advice: delete the last 'factors'

We have checked and corrected it as suggested.

g. You repeted the first factor with different understanding of the sentence: '(i), there are no studies on adaptive behavior in countries with transitional economies and strongly affected by climate change like Vietnam (Haile et al., 2013; Dat and Truong, 2020, Mai and Truong, 2022) (ii) there are few studies on adaptive behavior in countries with transition economies and strongly affected by climate change like Vietnam, (ii)'. Otherwise, the (ii) is mentioned twice.

We have checked and corrected it as suggested.
h. 'linked to agriculture. They are'. You are talking about AREA so you should change from 'YOU ARE' to 'It is'

We have checked and corrected it as suggested.
i. 'the introduction introduces'. Advide: The introduction SHOWS

We have checked and corrected it as suggested.
j. '(iii) results and discussion section presents research results on factors'. suggestion: research takeaways

We have checked and corrected it as suggested.
k. 'September to March of the following year, the average temperature is from 22 C - 23 C'. A comma is missing after 23°C

We have checked and corrected it as suggested.
l. last part before figure 1: 'and there is also moderate rain in December'. That was previously informed when you mentioned that between Sept - March it exists the rainy season.

We have checked and corrected it as suggested.
m. Figure 1: The names of the regions are not easily readable. We changed the graph for more detail and clear

We have changethe grapb as suggested.
n. Data collection and analysis: you use the word Collect many times, particularly in the 2nd paragraph.

We change the word ‘collect’ for more diverse.
o. 'Chau Hoa Phong Hoa and Duc Hoa Communes (Thanh, 2021).' A comma is missing to separe the regions.

We have checked and corrected it as suggested.
p. SOmetimes you write: analySys and sometimes analyZes. Are you writing in US or EN english?

We changed all to ‘analyse’
q. 'small family sizes.. The age'. DOUBLE PERIOD.

We changed to ‘The age of the household head was used as a measure of farming experience’.

r. '42.2 % and 31.3% of people questioned said that it was more than in 2018)' the initial ( is missing.

We checked and corrected.
s. 'In our study, the role of government is reflected through the institutional factors included in the model. Our results showed' I think it is better writtin

g in 3rd person.

We have checked and corrected it as suggested.

- Also there are many misconceptions. The main: 'Natural Disaster'. Please review the term: https://unu.edu/ehs/series/5-reasons-why-disasters-are-not-natural But also:-'Farmers' choice of adaptation strategies often depends on factors such as demographic and socio-economic characteristics': and technical/ community/ ancestral knowledge;

We have checked the suggested reference and from the lituerature revew, we added following paragraph as you suggestions:

‘…This study examines the factors influencing farmers' climate variability adaptation strategies in Vietnam which mighte include:

- Socio-economic factors: farm size, farm income, memberships in civic organizations, and farming experience.
- Demographic factors: family size and age.
- Institutional factors: access to credit services, markets, training, and climate change information….’
- 'As a country with a coastline of 3,260 km, climate change and rising sea'. In that phrase you are combining 2 different concepts: You should mention all the risks (rising sea) or the global concept of climate change.

We change to ‘As a country with a long coastline and high population density and coastal economic activities, Vietnam is considered one of the five countries most heavily affected by climate change.’

- Introduction: the lack of response resources is a low adaptative capacity. It you are taking on the natural resourses please specify the Sensibility.

We have added sensibility in the Introduction as suggested.

- -'drought adaptation strategy'. The adaptation strategy is FOR DROUGHT. The Drought is not an adaptation strategy.

We changed the sentencet to’ adaptation strategy for drought’ as comment.

- The prolonged heatwave and increasing seawater intrusion'. You mentioned that the impacts were drought and seawater intrusion. Please do not consider heatwaves and drought as synonims.

We changed it to heatway as suggested.

- Another thing to add, I think the full title could be Factors Influencing Farming HouseHolds´Climate Change Adaptation Strategies in Quang Binh, Central Vietnam, distinguishing it from the short one.

Thanks for the suggestion! We want this to be a case study in Vietnam and the implications of the study can be referenced for other coastal areas in Vietnam that are also affected by natural disasters in the context of climate change. Therefore, we have chosen a broader title.

- In the study, you were mentioning many times the awareness of Climate Change from the population but that question wasn´t included in the interview or is not formally analyzed, it appears to be an indirect conclusion

Thanks for the suggestion! We assessed people's awareness of natural disasters in the context of climate change including awareness of natural disaster trends in the last 5 years, intensity and frequency of natural disasters, and changes in the impact of natural disasters.

‘…..The study examined the awareness and adaptation strategies adopted by respondents to cope with with disasters in climate change context in local communities. The majority (85.23%) of selected households were aware of the existence of climate change and extreme events in their locality. Natural disasters frequently appeared in Tuyen Hoa in the last 5 years include floods, storms, saltwater intrusion, and drought. Frequency of occurrence of natural disasters compared to previous years according to people's objective point of view (drought, flood and saltwater intrusion), with corresponding percentages of 51.9%, 42.2 % and 31.3% of people questioned said that it was more than in 2018 while 63.4% thought that the frequency of storms was less. However, the intensity of each storm is much greater (for example, storm Wuitp appeared in October 2018) (Table 3).

As reported by households, in the study area, flood disasters often occur from August to October and last until November; drought and saltwater intrusion from April to July every year. These disasters and natural disasters affect agricultural activities, especially activities that target the cultivation of agricultural crops (rice, food crops, other crops...). Interviews said that the phenomenon of saltwater intrusion and drought is strongest from April to June and can last until July with huge damage to rice cultivation. Moreover, flooding often accompanies storms that occur in August, September, and October and can last until November (Table 3). People's awareness of climate change was quite similar to climate developments analysed in Vietnam national climate change and sea level rise scenarios (MONRE, 2020) (Figure 3).’

- To complete with the researching information, is that you implemented the interviews in the rainy season (Nov-Dec). Do you think that is also important to make interviews all along the year? Or at least between the 2 seasons you mentioned?

Due to limited resources, we only conducted the survey in one time slice - November and December (rainy season). However, because the local people have lived in the area for a long time, they have a clear experience of the impact of natural disasters in both the dry and rainy seasons. We will add this point to the research limitation section according to the reviewer's comments.

- Some references are missing, specifically in strong facts, such as:
  Introduction: I suggest to add FAO/ UNEP bibliography in the introduction with some figures. EG: 52% of global agricultural lands are now moderately to severely degrade (Source: UNEP FI)

The authors have added an avove fact in the Introduction section with UNEP references as suggested by the reviewer UNEP (2016). Land restoration key to human well-being. https://www.unep.org/news-and-stories/story/land-restoration-key-human-well-being.

- Introduction: which is the source to say 'the area that has suffered the most natural disasters'? It is a strong communication that should be back-up.

Thanks for the comment; we have added references to the above sentence, as follows:

‘…This is the area that has suffered the most natural disasters and negative impacts of climate change in Vietnam over the past 20 years, with people's livelihoods closely linked to agriculture (Mai and Truong, 2022)…’

- Data Collection and analysis: 'we first selected 3 communes of Tuyen Hoa District with a high proportion of farming households and severe vulnerability to climate change'. Which is the bibliography that determine the severe vulnerability? This should be mentioned as reference because it cannot be a public perception

We add the reference source for that sentence (Quang Binh People Committee, 2022).

- Figure 3: one unit is missing in the representation of the 1rst graphic.

We have checked and correct it.

Once again, the authors would like to thank the reviewer so much and hope to receive the consideration from the reviewer.
