# Supplementary material for: Factors influencing farming households’ climate change adaptation strategies in Central Vietnam
Source: PLoS One. 2025 Jul 10;20(7):e0328058. doi: 10.1371/journal.pone.0328058 (PMC12244606; doi:10.1371/journal.pone.0328058)
Supplement: Attachment — Submitted filename: Report to reviewers R2.docx [file pone.0328058.s004.docx]

**RESPONSE REPORT TO REVIEWER**

The authors would like to thank the reviewer so much for detailed comments and suggestions. These are very valuable comments that help the authors improve the article. The authors have fully received and revised the entire article according to the comments of the reviewer. Below is the author's detailed explanation and modification (in red text) according to each comment point of the editor and reviewers. We have also reviewed and improved the entire article in terms of rationale, novelties, research questions, academic contributions, references, discussions, language and format (Please see the revised paper for more detail). The authors hope to receive considerations by the editor and reviewer.

**Reviewer #3:**

**Comment 1:**

The authors mention VIF analysis but do not present a table of VIF values. Including a supplementary table with VIF values would enhance transparency and reproducibility.

**Response 1:**

Thank you so much for your valuable comment. The authors have added the VIF column in Table 3. Accordingly, the VIF values are all less than 2, so there is no multicollinearity in the analysis model.

**Comment 2:**

The manuscript uses “natural disaster,” a contested term in disaster studies. While the authors cite UNU-EHS to justify their use, it’s recommended to use “climate-related hazards” or “weather extremes” for conceptual accuracy.

**Response 2:**

Thank you for your valuable scientific comment. We have replaced ‘natural disasters’ with ‘climate-related hazards’ in the article as per your comment.

**Comment 3:**

Although much improved, minor grammatical issues remain (e.g., “trainning” should be “training”; “rikds” should be “risks” – though corrected in some parts, these still appear occasionally).

**Response 3:**

Thank you for your comment. We have rewritten many of the main contents of the revised paper in English academic style, and have checked and corrected all spelling and typos in the paper according to your comment.

**Comment 4:**

The authors have added 2023–2024 references, which is appreciated. However, more diversity in geographic scope (i.e., more studies from other vulnerable countries in Asia or Africa) would further strengthen the literature review.

**Response 4:**

Thank you for this important comment. The authors have added more recent references in Asia and Africa as suggested. Some examples include:

1. Kone S, Balde A, Zahonogo P, et al. A systematic review of recent estimations of climate change impact on agriculture and adaptation strategies perspectives in Africa. *Mitig Adapt Strateg Glob Change*. 2024;29:18.
2. Adam M, MacCarthy DS, Traoré PCS, et al. Which is more important to sorghum production systems in the Sudano‑Sahelian zone of West Africa: climate change or improved management practices? *Agric Syst*. 2020;185:102920.
3. Alemseged A, Getahun YS, Belete Y, et al. A systematic literature review on factors influencing climate-smart agriculture adoption among African farmers. *Front Environ Econ Rev*. 2023;1:1356335.
4. Cammarano D, Valdivia RO, Beletse YG, et al. Integrated assessment of climate change impacts on crop productivity and income of commercial maize farms in northeast South Africa. *Food Sec*. 2020;12:659–678.
5. Akum RA, Atiah WA, Amekudzi LK, et al. Climate variability and impacts on maize yield in Ghana: a systematic review. *Q J R Meteorol Soc*. 2022;148(742):185–198.
6. Batunga‑wayo P, Habarugira V, Vanclooster M, et al. Confronting climate change and livelihood: smallholder farmers’ perceptions and adaptation strategies in northeastern Burundi—a systematic review. *Reg Environ Change*. 2023;23(1):47.
7. Der Tambile E, Ramachandran VS, Rajendrakumar S, et al. Rural livelihoods sustainability in South Asia and Africa: a systematic review with bibliometric analysis. *Environ Sustain Indic*. 2024;
8. Sallah M, Antoniades G, Kourkafas GE, et al. Enabling climate change adaptation in coastal systems: a bibliometric review of empirical studies. *Earth’s Future*. 2023;1–22.
9. Datta P, Behera B, Rahut DB. Climate change and Indian agriculture: a systematic review of farmers’ perception, adaptation, and transformation. *Environ Dev Sustain*. 2024;26(3):4123–4142.
10. Sallah M, Antoniades G, Kourkafas GE, Tsiouri M. Enabling climate change adaptation in coastal systems: a bibliometric review of empirical studies. *Earth’s Future*. 2023;11(10):e2023EF004912.

**Comment 5:**

The updated version of Figure 3 still lacks units on the first graph axis.

**Response 5**

The authors have checked and added according to the comment in Figure 3.

**Comment 6:**

Please use the Vancouver referencing style. Include continuous line numbering for the text.

**Response 6:**

Thank you so much. We have adjusted and used the Vancouver referencing style in the revised paper. The authors have also added line numbering for the text as suggested.

**Comment 7:**

Conclusions- Tie the findings more clearly to policy — e.g., what type of training interventions are needed? Who should provide credit (state, microfinance)? Should salt-tolerant rice be subsidised?

**Response 6:**

We thank the reviewer for this insightful comment. In response, we have revised the **Recommendations** section to explicitly link the empirical findings to specific policy actions and institutional responsibilities. In particular, we now detail the types of training interventions needed, identify key stakeholders for credit provision, and discuss the potential role of targeted subsidies for salt-tolerant rice. These additions aim to improve the practical relevance and policy utility of the study’s conclusions. For example:

***Design Tailored Training Programs Focused on Climate-Resilient Agricultural Practices***

Based on the significant role of training participation in promoting adaptation behaviors, especially for technically demanding strategies such as salt-tolerant rice cultivation and lotus-fish integration, it is recommended that training programs be customized to meet local needs. These should go beyond general awareness-raising and focus on hands-on, demonstration-based modules covering soil salinity management, integrated farming techniques, and irrigation scheduling. Training should be organized regularly at the commune or village level and facilitated through a partnership between the Department of Agricultural Extension, local universities, and farmer cooperatives.

***Expand Access to Credit through Multi-Tiered Financial Systems***

Given the finding that access to credit modestly improves adoption of capital-intensive strategies such as shrimp and lotus-fish farming, policy should promote the development of a multi-tiered rural finance system. This could include expanding the outreach of the Vietnam Bank for Social Policies (VBSP) in parallel with mobilizing microfinance institutions (MFIs) and credit unions. Priority should be given to low-interest, climate-resilient loans targeting smallholders in salinity-prone regions, especially female-headed households or members of local civic organizations.

***Introduce Targeted Subsidies for Salt-Tolerant Rice Varieties***

As salt-tolerant rice varieties were among the most widely adopted strategies but still showed sensitivity to land characteristics and income levels, we recommend the introduction of targeted input subsidies. These should focus on reducing the cost burden of seed acquisition and initial training for poor and medium-income households. The subsidies could be implemented through voucher-based schemes coordinated by provincial agricultural departments, with monitoring mechanisms to ensure proper targeting and transparency. In parallel, public investment in research and development (R&D) of high-yield, climate-resilient rice strains should be continued and linked to extension services.

*Once again, the authors would like to thank the reviewer so much and hope to receive the consideration and acceptance from the reviewer!*
